# The Suppressive Effects of Cinnamomi Cortex and Its Phytocompound Coumarin on Oxaliplatin-Induced Neuropathic Cold Allodynia in Rats

**DOI:** 10.3390/molecules21091253

**Published:** 2016-09-20

**Authors:** Changmin Kim, Ji Hwan Lee, Woojin Kim, Dongxing Li, Yangseok Kim, Kyungjin Lee, Sun Kwang Kim

**Affiliations:** 1 Department of Physiology, College of Korean Medicine, Kyung Hee University, 26 Kyunghee-daero, Dongdamoon-gu, Seoul 02447, Korea; ckdals4302@naver.com (C.K.); thasnow@gmail.com (W.K.); yskim1158@khu.ac.kr (Y.K.); 2 Department of Science in Korean Medicine, Graduate School, Kyung Hee University, 26 Kyunghee-daero, Dongdamoon-gu, Seoul 02447, Korea; mibdna@gmail.com; 3 Department of Anesthesiology, Renji Hospital, School of Medicine, Shanghai Jiaotong University, 573 Xujiahui Rd., Dapiqiao, Huangpu Qu, Shanghai 200025, China; leedongxing@naver.com; 4 Department of Herbology, College of Korean Medicine, Kyung Hee University, 26 Kyunghee-daero, Dongdamoon-gu, Seoul 02447, Korea

**Keywords:** Cinnamomi Cortex, cold allodynia, coumarin, glia, spinal cord, pro-inflammatory cytokines

## Abstract

Oxaliplatin, a chemotherapy drug, induces acute peripheral neuropathy characterized by cold allodynia, spinal glial activation and increased levels of pro-inflammatory cytokines. Herein, we determined whether Cinnamomi Cortex (C. Cortex), a widely used medicinal herb in East Asia for cold-related diseases, could attenuate oxaliplatin-induced cold allodynia in rats and the mechanisms involved. A single oxaliplatin injection (6 mg/kg, i.p.) induced significant cold allodynia signs based on tail immersion tests using cold water (4 °C). Daily oral administration of water extract of C. Cortex (WECC) (100, 200, and 400 mg/kg) for five consecutive days following an oxaliplatin injection dose-dependently alleviated cold allodynia with only a slight difference in efficacies between the middle dose at 200 mg/kg and the highest dose at 400 mg/kg. WECC at 200 mg/kg significantly suppressed the activation of astrocytes and microglia and decreased the expression levels of IL-1β and TNF in the spinal cord after injection with oxaliplatin. Furthermore, oral administration of coumarin (10 mg/kg), a major phytocompound of C. Cortex, markedly reduced cold allodynia. These results indicate that C. Cortex has a potent anti-allodynic effect in oxaliplatin-injected rats through inhibiting spinal glial cells and pro-inflammatory cytokines. We also suggest that coumarin might play a role in the anti-allodynic effect of C. Cortex.

## 1. Introduction

Oxaliplatin, a third-generation platinum-based chemotherapy drug, displays anti-tumor activity against a wide range of tumors, including ovarian, breast, lung, and advanced colorectal cancers [1,2,3]. Unlike other platinum compounds (e.g., cisplatin and carboplatin), oxaliplatin rarely exhibits nephrotoxicity or hematotoxicity [4]. However, even a single injection of oxaliplatin can cause peripheral neuropathy manifesting as paresthesia and dysesthesia in the extremities. This peripheral neuropathy can be triggered or enhanced by exposure to cold [5,6]. The accurate mechanism involved in oxaliplatin-induced peripheral neuropathy remains unclear. An effective treatment method for neuropathic allodynia needs to be developed [7].

Glial cells are known to play an important role in neuropathic pain [8,9]. Once damage in the periphery or the dorsal horn of the spinal cord occurs, glial cells such as microglia and astrocytes become activated [10,11] and proliferate in number [8,12]. Recently, some articles have demonstrated that pharmacological treatments can effectively reduce neuropathic pain by preventing glial cell activation [13,14]. In an oxaliplatin-induced neuropathic pain model, the decrease of augmented spinal astrocytes and microglia by fluorocitrate and minocycline has been reported to attenuate pain [10]. Glia cells contribute to neuropathic pain by releasing various nociceptive mediators including pro-inflammatory cytokines, such as IL-1β and TNF [15,16,17]. Moreover, in our previous experiment conducted with oxaliplatin-induced neuropathic pain in rats, increased activation of spinal astrocytes and microglia [18] as well as increased levels of spinal TNF were observed [19].

Cinnamomi Cortex (C. Cortex; *Cinnamomum cassia* Presl in family Lauraceae) is classified as a medicinal herb for treating various cold related diseases such as common cold and influenza in oriental medicine. Using various human and animal models, extracts of C. Cortex have been found to be able to effectively attenuate influenza virus [20], inflammations [21], human platelet aggregation and arachidonic acid metabolism [22]. In our previous study [18], Gyejigachulbu-tang (GBT), a decoction comprising seven different medicinal herbs (Cinnamomi Cortex, Paeoniae Radix, Atractylodis Rhizoma, Zizyphi Fructus, Glycyrrhizae Radix, Zingiberis Rhizoma, and Aconiti Tuber) could effectively attenuate oxaliplatin-induced neuropathic pain. GBT also suppressed the activation of spinal glia and the up-regulation of pro-inflammatory cytokines. However, which medicinal herb plays a major role in the analgesic effect of GBT has not yet been determined.

Accordingly, in this study, we investigated whether C. Cortex, when administered alone, could attenuate oxaliplatin-induced cold allodynia in rats. We also examined whether C. Cortex could modulate the activation of astrocytes and microglia in the spinal cord and suppress the upregulation of pro-inflammatory cytokines such as IL-1β and TNF after oxaliplatin injection. Finally, we assessed whether coumarin, a major phytocompound of C. Cortex, could alleviate oxaliplatin-induced neuropathic pain.

## 2. Results

### 2.1. Suppresive Effect of WECC on Oxaliplatin-Induced Cold Allodynia in Rats

**Figure 1:**
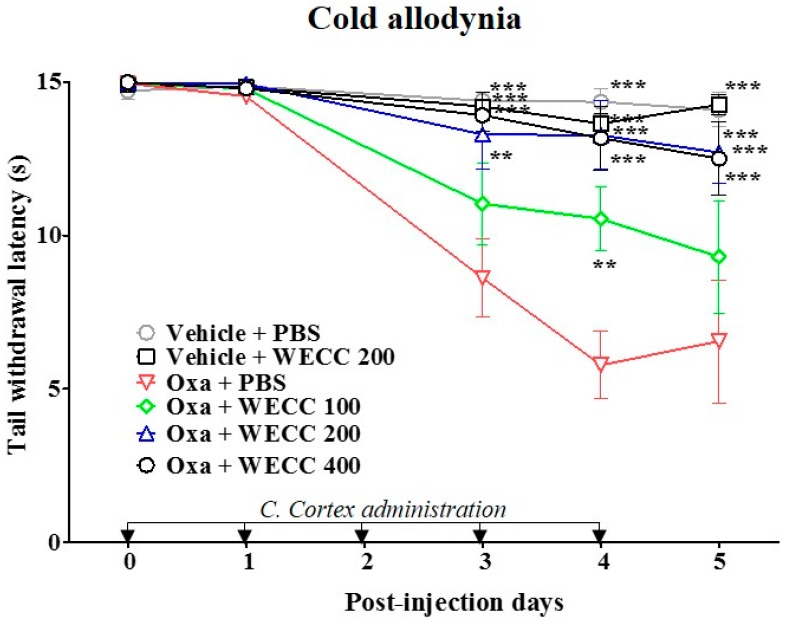
Effect of WECC in relieving oxaliplatin-induced cold allodynia in rats. Animals were randomly divided into five groups (*n* = 6 per group). Oxaliplatin (Oxa) or 5% glucose (Vehicle) was administered intraperitoneally at day 0. WECC (100, 200, and 400 mg/kg) or PBS was administered orally for five consecutive days after injection of oxaliplatin or vehicle control. Data are presented as mean ± S.E.M.; ** *p* < 0.01, *** *p* < 0.001 vs. Oxa + PBS by two-way ANOVA followed by Bonferroni’s post-test.

A single injection of oxaliplatin (6 mg/kg, intraperitoneal; i.p.) induced cold allodynia in rats whereas vehicle control (5% glucose) did not. Cold allodynia was assessed using tail immersion test by measuring tail withdrawal latency (TWL) to cold water (4 °C) stimuli [23]. As shown in Figure 1, the TWL in oxaliplatin-injected rats was significantly decreased compared to that in vehicle-injected control rats from day 3 to day 5. To evaluate the suppressive effect of C. Cortex on oxaliplatin-induced cold allodynia, water extract of C. Cortex (WECC) at three different doses (100, 200, and 400 mg/kg) was orally administered every day for five consecutive days after the oxaliplatin injection. Behavioral tests were performed before oxaliplatin injection and every 24 h after the administration of WECC or phosphate-buffered saline (PBS). WECC dose-dependently attenuated oxaliplatin-induced cold allodynia. The lowest dose of WECC at 100 mg/kg showed a significant effect only at day 4, whereas WECC at 200 and 400 mg/kg had potent analgesic effects from three to five days after the oxaliplatin injection with only slight difference in efficacies between the two doses (Figure 1). In addition, WECC at 200 mg/kg showed a significant suppressive effect against mechanical allodynia at day 5 (Figure S1).

### 2.2. Inhibitory Effect of WECC Treatment on Activation of Spinal Astrocytes

**Figure 2:**
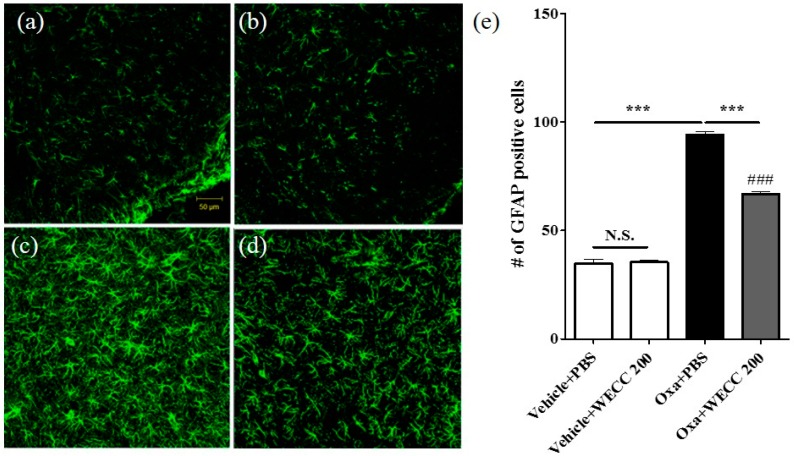
Immunohistochemical analyses of spinal astrocytes. Representative images of GFAP positive cells (astrocytes) in the spinal dorsal horn of Vehicle + PBS (**a**); Vehicle + 200 mg/kg WECC (**b**); Oxa + PBS (**c**); and Oxa + 200 mg/kg WECC (**d**) groups; (**e**) Quantification results of GFAP positive cells in the four groups. *n* = 6 per group. Data are presented as mean ± SEM. N.S.: non-significant; *** *p* < 0.001 vs. indicated group; ### *p* < 0.001 vs. Vehicle + PBS by one-way ANOVA followed by Bonferroni’s post-test.

At the end of behavioral experiments, spinal cord sections were obtained from animals in Vehicle + PBS, Vehicle + 200 mg/kg WECC, Oxaliplatin + PBS, and Oxaliplatin + 200 mg/kg WECC groups and processed for immunohistochemical analyses of astrocytic activation (Figure 2). Statistical differences in the number of astrocytes (GFAP positive cells) in the spinal dorsal horn laminae I–II were found between oxaliplatin-injected rats and vehicle-injected rats (Vehicle + PBS vs. Oxa + PBS; *p* < 0.001). WECC at 200 mg/kg failed to change the number of spinal astrocytes in vehicle-injected rats (Vehicle + PBS vs. Vehicle + 200 mg/kg WECC; *p* > 0.05). However, it significantly decreased the number of spinal astrocytes in oxaliplatin-injected rats (Oxa + PBS vs. Oxa + 200 mg/kg WECC; *p* < 0.001).

### 2.3. Inhibitory Effect of WECC Treatment on Spinal Microglia Activation

**Figure 3:**
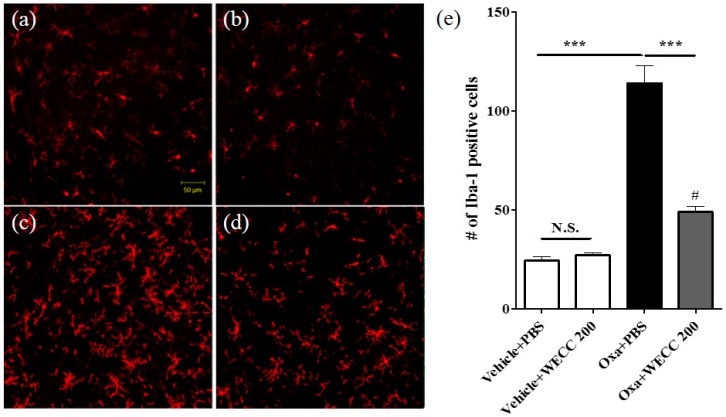
Immunohistochemical analyses of spinal microglia. Representative images of Iba-1 positive cells (microglia) in the spinal dorsal horn of Vehicle + PBS (**a**); Vehicle + 200 mg/kg WECC (**b**); Oxa + PBS (**c**); and Oxa + 200 mg/kg WECC (**d**) groups; (**e**) Quantification results of Iba-1 positive cells in the four groups. *n* = 6 per group. Data are presented as mean ± S.E.M. N.S.: non-significant; *** *p* < 0.001 vs. indicated group; # *p* < 0.05 vs. Vehicle + PBS by one-way ANOVA followed by Bonferroni’s post-test.

After the final assessment of cold allodynia, the spinal cord sections from animals in vehicle + PBS, vehicle + 200 mg/kg WECC, oxaliplatin + PBS, and oxaliplatin + 200 mg/kg WECC groups were obtained for histological examination (Figure 3). Statistical differences in the number of microglia (Iba-1 positive cells) in the spinal dorsal horn laminae I–II were found between oxaliplatin-injected rats and vehicle-injected rats (Vehicle + PBS vs. Oxa + PBS; *p* < 0.001). WECC failed to change the number of spinal microglia in vehicle-injected rats (Vehicle + PBS vs. Vehicle + 200 mg/kg WECC; *p* > 0.05). However, it significantly decreased the number of spinal microglia in oxaliplatin-injected rats (Oxa + PBS vs. Oxa + 200 mg/kg WECC; *p* < 0.001).

### 2.4. Modulatory Effect of WECC on Pro-Inflammatory Cytokines IL-1β and TNF

**Figure 4:**
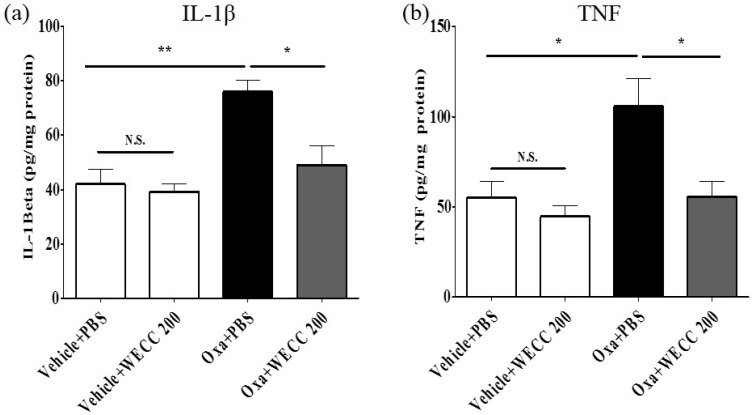
Inhibitory effect of WECC on spinal pro-inflammatory cytokines. Levels of IL-1β (**a**) and TNF (**b**) in the spinal cord were measured with ELISA. Vehicle and oxaliplatin were administered intraperitoneally. PBS and 200 mg/kg of WECC were administered orally. *n* = 6 per group. Data are presented as mean ± SEM. N.S.: non-significant; * *p* < 0.05; ** *p* < 0.01 by one-way ANOVA followed by Bonferroni’s post-test.

To investigate whether C. Cortex could modulate pro-inflammatory cytokines IL-1β and TNF in the spinal cord, an enzyme-linked immunosorbent assay (ELISA) was used to measure the levels of cytokines IL-1β and TNF (Figure 4). A single oxaliplatin injection markedly increased spinal IL-1β levels compared to vehicle control injection (Figure 4a, Oxa + PBS vs. Vehicle + PBS; *p* < 0.01). Administration of WECC at 200 mg/kg significantly reduced spinal IL-1β levels compared to PBS administration in oxaliplatin-injected rats (Figure 4a, Oxa + PBS vs. Oxa + 200 mg/kg WECC; *p* < 0.05). Oxaliplatin also significantly increased spinal TNF levels compared to vehicle control (Figure 4b, Oxa + PBS vs. Vehicle + PBS; *p* < 0.05). The levels of TNF were significantly reduced by WECC at 200 mg/kg (Figure 4b, Oxa + PBS vs. Oxa + 200 mg/kg WECC; *p* < 0.05). WECC failed to significantly change the levels of spinal IL-1β or TNF in vehicle-injected rats (Figure 4a or Figure 4b, Vehicle + PBS vs. Vehicle + 200 mg/kg; *p* > 0.05). These results indicate that C. Cortex can reduce the levels of pro-inflammatory cytokines IL-1β and TNF in the spinal cord increased by injection of oxaliplatin. 

### 2.5. Qualitative and Quantitative Analysis of Three Chemicals in WECC

**Figure 5:**
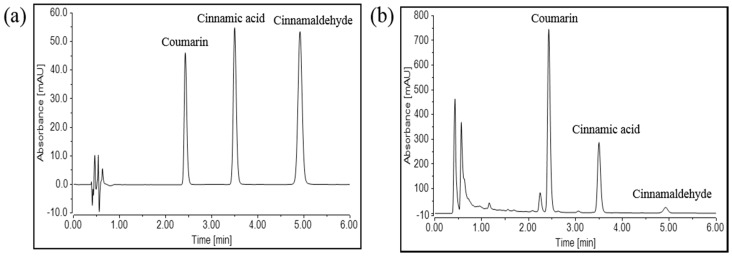
Quantification of chemicals in WECC using UHPLC. UHPLC chromatograms of three standards (**a**) and WECC (**b**). The three peaks represented coumarin (2.430 min, *y* = 0.0013*x* + 8.7147, *r*^2^ = 0.9987), cinnamic acid (3.500 min, *y* = 0.0047*x* − 0.5087, *r*^2^ = 0.9992), and cinnamaldehyde (4.922 min, *y* = 0.0058*x* − 0.0110, *r*^2^ = 1.0000) sequentially.

The retention times of coumarin, cinnamic acid, and cinnamaldehyde in WECC were 2.430, 3.500, and 4.922 min, respectively (Figure 5a). The regression equation of the three compounds and their correlation coefficients (*r*^2^) were estimated based on the plots of peak-area (*y*) versus concentration (*x*). For coumarin, the regression equation was *y* = 0.0013*x* + 8.7147 (*r*^2^ = 0.9987). For cinnamic acid, it was *y* = 0.0047*x* – 0.5087 (*r*^2^ = 0.9992). For cinnamaldehyde, it was *y* = 0.0058*x* − 0.0110 (*r*^2^ = 1.0000). The contents of coumarin, cinnamic acid, and cinnamaldehyde in WECC were 1.468%, 0.226%, and 0.027%, respectively (Figure 5b).

### 2.6. Anti-Allodynic Effect of Coumarin on Oxaliplatin-Induced Cold Allodynia in Rats

**Figure 6:**
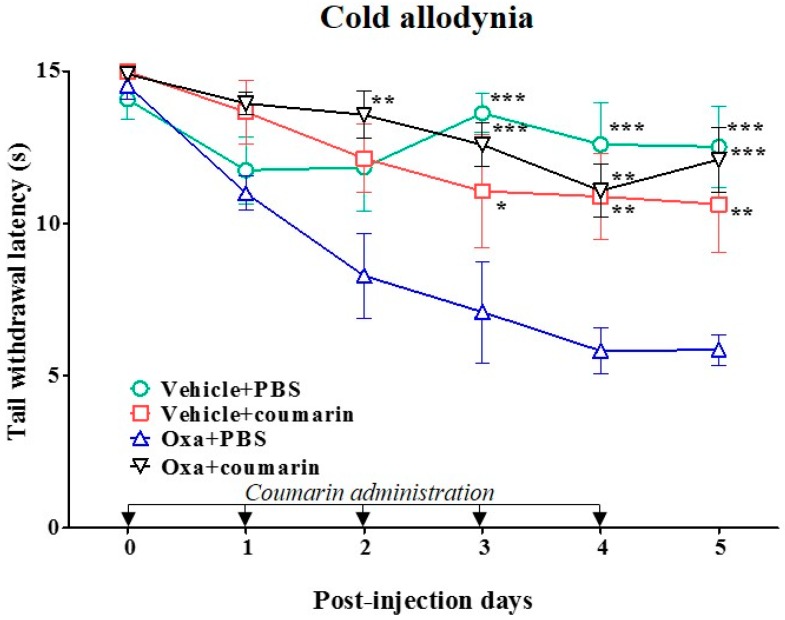
Anti-allodynic effect of coumarin on oxaliplatin-induced cold allodynia in rats. Animals were randomly divided into four groups (*n* = 6 per group). Oxaliplatin or vehicle (5% glucose) was administered intraperitoneally on day 0. Coumarin (10 mg/kg) or PBS was administered orally for five consecutive days after injection with oxaliplatin or vehicle control. Data are presented as mean ± S.E.M.; * *p* < 0.05, ** *p* < 0.01, *** *p* < 0.001 vs. Oxa + PBS by two-way ANOVA followed by Bonferroni’s post-test.

A previous study [24] reported that coumarin at 10 mg/kg has the most effective antinociceptive action, as our preliminary study has confirmed in a rat model of oxaliplatin-induced cold allodynia. In the present study, we thus tested the anti-allodynic effect of 10 mg/kg coumarin, a major chemical component of C. Cortex, by conducting tail immersion test. Results are shown in Figure 6. Coumarin (10 mg/kg) orally administered in vehicle (5% glucose) injected rats did not have any significant anti-allodynic effect (Vehicle + coumarin vs. Vehicle + PBS; *p* > 0.05). However, the same dose of coumarin significantly increased the TWL compared to PBS control in oxaliplatin-injected rats (Oxa + coumarin vs. Oxa + PBS; *p* < 0.001). The significant anti-allodynic effect of coumarin started at day 3 and lasted until day 5 after a single injection with oxaliplatin. In addition, coumarin slightly increased mechanical threshold in oxaliplatin-injected rats, although no significant difference was observed between the coumarin group and PBS control group (Figure S1).

## 3. Discussion

Oxaliplatin is a widely used chemotherapeutic agent. About 85% to 95% of oxaliplatin-treated patients rapidly develop significant pain without motor dysfunction, peaking at the first 24–48 h following the oxaliplatin injection [25,26]. This side effect can limit the use of this drug and lead to cessation of the treatment. However, the mechanism of oxaliplatin-induced peripheral neuropathy is not clearly understood yet. Analgesics currently used as first-line treatment also have side effects such as sedation, dizziness, and cardiac complications [27,28]. Thus, a novel treatment method is urgently needed.

Recently, deactivation of spinal astrocytes or microglia has been considered as a new pharmacological target for neuropathic pain relief [10,29]. In various models of neuropathic pain, glial activation has been reported to contribute to neuropathic pain by releasing pro-inflammatory cytokines [30]. Once activated after peripheral nerve injury, glia cells such as astrocytes and microglia can release a plethora of pro-inflammatory mediators such as IL-1β and TNF [31,32] involved in the process of nociceptor hypersensitivity [33]. In an injured sciatic nerve animal model, mRNA and protein levels of IL-1β and TNF are rapidly up-regulated, and mice lacking both IL-1 type 1 receptor and TNF type 1 receptor have lower nociceptive sensitivity compared to wild-type littermates after injury [34]. IL-1β up-regulation following nerve injury contributes to pain hypersensitivity through mediating the induction of Cox-2 in the central nervous system (CNS) [35]. TNF, a major pro-inflammatory cytokine, has been associated with both immediate and ongoing stages of neuropathic pain. TNF applications are associated with induction of thermal hyperalgesia and mechanical allodynia [36,37]. Moreover, intrathecal injection of minocycline or fluorocitrate that decreases the activation of microglia or astrocytes effectively attenuates oxaliplatin-induced neuropathic pain [10].

In our previous articles [18,19], we have shown that spinal glia and pro-inflammatory cytokines are up-regulated after a single injection with oxaliplatin. We also demonstrated that GBT, a mixture comprising seven different medicinal herbs including C. Cortex based on the *Sang Han Lun* and later modified in Japan [38], could alleviate neuropathic pain induced by oxaliplatin injection through suppressing the activation of spinal astrocytes and microglia and decreasing the levels of spinal TNF. However, a traditional formula of GBT contains Cinnamomi ramulus (C. Ramulus) instead of C. Cortex. Both C. Cortex and C. Ramulus originate from cinnamon. They are commonly used as traditional medicine for treating dyspepsia, gastritis, blood circulation issues, and inflammatory disease [39]. C. Ramulus is the twig of cinnamon while C. Cortex is the bark of cinnamon. Although the analgesic efficacies of C. Cortex and C. Ramulus for pain have been reported in several articles [40,41,42], we found that C. Ramulus did not significantly attenuate cold allodynia induced by oxaliplatin injection (Figure S2). In oriental medicine, different parts of the same plant are considered as distinct medicinal herbs having separate characteristics [43].

In this study, we clearly demonstrated that C. Cortex, one traditional medicinal herb generally used to treat cold related diseases such as common cold and influenza, could alleviate cold allodynia induced by a single injection of oxaliplatin in rats. Five consecutive oral administrations of WECC dose-dependently attenuated cold allodynia, suppressed the activation of spinal astrocytes and microglia, and inhibited the up-regulation of spinal pro-inflammatory cytokines IL-1β and TNF in oxaliplatin-injected rats. These results suggest that C. Cortex might play an important role in the inhibitory effect of GBT on oxaliplatin-induced cold allodynia via suppressing spinal glia and pro-inflammatory cytokines. C. Cortex contains various phytocompounds such as coumarin, cinnamic acid, and cinnamaldehyde [44]. Since previously published articles have reported that coumarin can exert anti-nociceptive action in different types of pain [24,45,46], we assessed the efficacy of coumarin on oxaliplatin-induced cold allodynia. Our results revealed that coumarin could effectively attenuate oxaliplatin-induced cold allodynia, suggesting that coumarin might play a role in the anti-allodynic effect of C. Cortex in a rat model of oxaliplatin-induced neuropathic cold allodynia. 

Although some articles have reported that activation of spinal glial cells occurred throughout the spinal cord, in our study [47], we focused on glial activation in lamina I and II because it is well known that this area is important for pain sensation as the primary afferent nociceptive neurons form synapses to the secondary afferent ones in this area. Furthermore, other studies have also observed the activation of microglia and astrocytes in this area to assess whether their compound or drug could modulate the sensory transmission by decreasing the activation of spinal glial cells in neuropathic pain rats [48]. Because we used tail immersion tests for evaluating neuropathic cold allodynia, we assessed glial activation not only in the lumbar spinal cord (Figure 2 and Figure 3), but also in the sacral spinal cord at the S1/S2 level that mainly innervates the tail (Figures S3 and S4). In these supplementary data, activation of astrocytes and microglia in the sacral spinal cord after oxaliplatin injection did not differ from that in the lumbar spinal cord. We confirmed that WECC at 200 mg/kg markedly suppressed oxaliplatin-induced activation of astrocytes and microglia in the sacral spinal cord. Coumarin also significantly inhibited such glial activation in the sacral spinal cord.

In conclusion, our results demonstrate that C. Cortex could be an effective medicinal herb to attenuate oxaliplatin-induced cold allodynia, by suppressing the activation of spinal astrocytes and microglia and inhibiting the increase of spinal levels of IL-1β and TNF. Our data also showed that coumarin, a major phytocompund of C. Cortex, might play a role in the anti-allodynic effect of C. Cortex. Additional animal studies using different neuropathic pain models, such as peripheral nerve injury and diabetic neuropathy, and clinical trials may be required to expand the therapeutic use of C. Cortex and/or coumarin.

## 4. Materials and Methods

### 4.1. Animals

Young adult male Sprague-Dawley rats (200–250 g, 7 weeks old) (Daehan Biolink, Chungbuk, Korea) were housed in cages (3–4 rats per cage) with water and food available *ad libitum*. The room was maintained with a 12 h-light/dark cycle (light cycle: 08:00–20:00, dark cycle: 20:00–08:00) and kept at 23 ± 2 °C. All animals were acclimated in their cages for one week prior to any experiment. All procedures involving animals were approved by the Institutional Animal Care and Use Committee of Kyung Hee University (KHUASP(SE)-15-088) and were conducted in accordance with the guidelines of the International Association for the Study of Pain [49].

### 4.2. Oxaliplatin Administration

As described previously [50], oxaliplatin (Sigma, St. Louis, MO, USA) was dissolved in a 5% glucose (Sigma) solution at a concentration of 2 mg/mL. It was intraperitoneally administered at 6 mg/kg [51]. The same volume of 5% glucose solution was intraperitoneally injected as vehicle control.

### 4.3. Behavior Test

To determine whether cold allodynia was induced, tail immersion test was carried out as described previously [23]. Briefly, each animal was immobilized in a plastic holder and its tail was drooped for proper application of cold water stimuli. Rats were adapted for one hour in the holder for two days before starting the behavioral tests. The tail was immersed in 4 °C water, and tail withdrawal latency (TWL) was measured with a cut-off time of 15 s. The cold immersion test was repeated six times at five minutes intervals. The average latency was taken as a measure for the severity of cold allodynia. A shorter TWL was interpreted as more severe allodynia. Our previous studies [18,23] showed that a significant allodynic behavior was induced from three days which lasted up to seven days after a single injection with oxaliplatin (6 mg/kg, i.p.). The behavior test was performed in blind condition.

### 4.4. Experimental Schedule and Grouping and Treatment of WECC

In this study, dried C. Cortex (500 g) was extracted with distilled water at 100 °C for 1 h. After filtration, the water extracts were evaporated using a rotary evaporator at 60 °C and lyophilized to yield 25.319 g of crude extract. Rats were orally injected with water extract of C. Cortex (WECC) diluted in PBS at 100, 200, or 400 mg/kg/day for five days after oxaliplatin injection. The doses of WECC used in this study were similar to those of other herbal formula used in our previous study [18,52] and those of Aconiti tuber in other’s study [48]. Rats were randomly divided into the following six groups (*n* = 6 per group for cold): (1) Vehicle + PBS (i.p. injection of 5% glucose solution + daily oral administration of PBS); (2) Vehicle + 200 mg/kg WECC (i.p. injection of 5% glucose solution + daily oral administration of 200 mg/kg WECC); (3) Oxa + PBS (i.p. injection of oxaliplatin + daily oral administration of PBS); (4) Oxa + 100 mg/kg WECC (i.p. injection of oxaliplatin + daily oral administration of 100 mg/kg WECC); (5) Oxa + 200 mg/kg WECC (i.p. injection of oxaliplatin + daily oral administration of 200 mg/kg WECC); and (6) Oxa + 400 mg/kg WECC (oxaliplatin injection + daily oral administration of 400 mg/kg WECC). On day 0, tail immersion test was performed at 10:00–12:00 and then oxaliplatin (6 mg/kg) or vehicle (5% glucose solution) was intraperitoneally injected followed by oral administration of WECC or PBS at 12:00–13:00. From day 1 to day 4, WECC or PBS administration was daily performed at 12:00–13:00 after behavioral test was conducted at 10:00–12:00. On day 5, only behavior test was performed at 10:00–12:00.

### 4.5. Immunohistochemistry

The animals anesthetized with isoflurane were transcardially perfused with 0.1 M PBS and fixed with a freshly prepared solution consisting of 4% paraformaldehyde (PFA) in 0.1 M phosphate buffer (pH 7.4). The lumbar spinal cord segment at the L4/L5 level and the sacral spinal cord segment at the S1/S2 level were extracted and post-fixed overnight in 4% PFA and transferred into 30% sucrose. Post-fixed lumbar segments were embedded in optimal cutting temperature (OCT) compound (Sakura Finetek, Tokyo, Japan) and kept in a box filled with dry ice. Samples of lumbar spinal cord segments were sectioned at 20 μm thickness using cryostat (Microm HM 505N; Thermo Fisher Scientific, Waltham, MA, USA). These sections were collected in 0.1 M PBS at 4 °C, mounted onto glass slides (Matsunami, Osaka, Japan), and incubated in 0.2% Triton X-100 in 0.5% bovine serum albumin (BSA; BOVOGEN biologics, East Keilor, Australia) solution at room temperature for 1 h. After rinsing the glass slides with 0.5% BSA solution, double immunostaining using primary antibodies raised in different species was performed. These sections were incubated overnight at 4 °C with mixed primary antibodies: mouse anti-glial fibrillary acidic protein (GFAP 1:500; Millipore, Ramona, CA, USA) and rabbit anti-Iba 1 (1:500; Wako, Osaka, Japan). After 0.5% BSA solution rinses, these sections were incubated with secondary antibodies: anti-mouse and anti-rabbit-immunoglobulin G (IgG) labeled with Alexa Fluor 488 and Alexa Fluor 546 (1:200; Invitrogen, Carlsbad, CA, USA) at room temperature in dark for 1 h. Confocal laser microscope (LSM 5 Pascal, Zeiss, Oberkochen, Germany) was used to obtain immunohistochemical images. To determine the efficacy of WECC on oxaliplatin-induced spinal glial activation, the number of GFAP and Iba-1 positive cells were manually counted and averaged in both sides of the spinal dorsal horn laminae I and II. Six spinal cord sections from each animal were used. Cell counting procedure was performed in blind condition.

### 4.6. ELISA

To determine whether WECC administration decreased the levels of TNF or IL-1β in the spinal cord, the levels of each cytokine were measured by enzyme linked immunosorbent assay (ELISA). Isoflurane anesthetized animal were transcardially perfused with 0.1 M PBS, the L4/L5 segments of the spinal cord were exposed from the vertebral column via laminectomy and identified by tracing the dorsal roots from their respective dorsal root ganglia (DRG). Collected tissues were stored in 1 mL RIPA buffer (Thermo Scientific) containing protease inhibitor cocktail (Roche, Basel, Switzerland). Samples were assayed using a commercial rat TNF ELISA kit (BD OptEIA Set Rat TNF, BD biosciences, San Jose, CA, USA) and mouse IL-1β ELISA kit (BD OptEIA Set mouse IL-1β, BD biosciences) following the manufacturer’s protocols. Optical density (O.D.) was measured at 450 nm with λ correction of 570 nm using a microplate reader (Tecan). Total amounts of protein in samples were measured using Bio-Rad protein assay kit (Bio-Rad, Hercules, CA, USA). Samples and standards were run in duplicate. All results were normalized to the total amount of protein in each sample.

### 4.7. Ultra High-Performance Liquid Chromatography (UHPLC) Analysis

Chromatographic analysis was performed using a Thermo Scientific Vanquish UHPLC system (Thermo Fisher Scientific) with Zorbax Eclipse Plus C18 (2.1 × 100 mm, 1.8 μm) column (Agilent Technology, Santa Clara, CA, USA). Mobile phase A (0.005% FA in water (*v*/*v*)) and mobile phase B (100% ACN) were operated with a gradient elution at a flow rate of 0.5 mL/min as follows: 25% B (0–6 min) → 100% B (6–6.5 min) → 100% B (6.5–9.5 min) → 25% B (9.5–10 min) → 25% B (10–13 min). The column temperature was set at 40 °C. Cinnamic acid (1 mg/mL), coumarin (1 mg/mL), and cinnamaldehyde (1 mg/mL) were dissolved in methanol and used as standards for the qualitative and quantitative analysis of WECC (2 mg/mL). Sample injection volume was 2 µL. Absorbance of column eluent was monitored with a UV spectrometer at wavelength of 280 nm.

### 4.8. Statistical Analysis

All data are presented as mean ± S.E.M (standard error of the mean). Statistical analysis and graphic works were done with Prism 5.0 (Graph Pad Software, La Jolla, CA, USA). The sample size was determined based on our previously conducted experiments [18,23]. One-way or two-way analysis of variance (ANOVA) followed by Bonferroni’s post hoc test was used for comparisons between groups. In all cases, *p* < 0.05 was considered as statistically significant.

## Supplementary Materials

Supplementary materials can be accessed at: http://www.mdpi.com/1420-3049/21/9/1253/s1.
