# Supplementary material for: The Suppressive Effects of Cinnamomi Cortex and Its Phytocompound Coumarin on Oxaliplatin-Induced Neuropathic Cold Allodynia in Rats
Source: Molecules. 2016 Sep 20;21(9):1253. doi: 10.3390/molecules21091253 (PMC6274362; doi:10.3390/molecules21091253)
Supplement: Supplementary file 1 [file molecules-21-01253-s001.pdf]

Supplementary Materials: Suppressive Effects of Cinnamomi Cortex and Its Phytocompound Coumarin on Oxaliplatin-Induced Neuropathic Cold Allodynia in Rats

Changmin Kim, Ji Hwan Lee, Woojin Kim, Dongxing Li, Yangseok Kim, Kyungjin Lee and Sun Kwang Kim

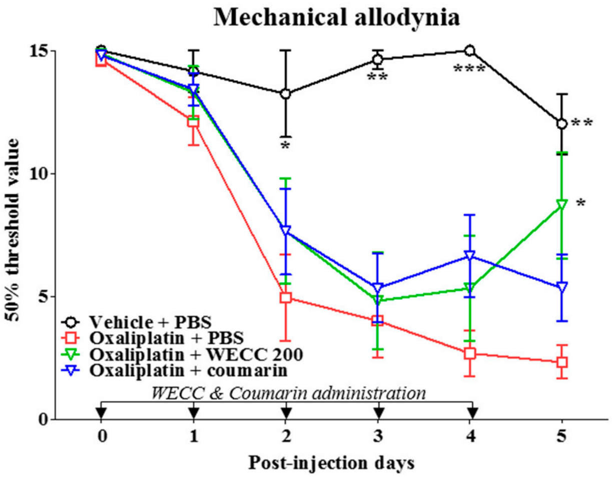

Figure S1. Effects of WECC and Coumarin on oxalipaltin-induced mechanical allodynia in rats. Animals were randomly divided into 4 groups (*n* = 6/group), Vehicle + PBS, Oxaliplatin + PBS, Oxaliplatin + WECC 200, Oxaliplatin + coumarin. Oxaliplatin or vehicle (5% glucose) was administered intraperitoneally on day 0. WECC (200 mg/kg), coumarin (10 mg/kg) or PBS was administered orally for five consecutive days after an oxaliplatin or vehicle injection. Data are presented as mean ± S.E.M.; * $p < 0.05$; ** $p < 0.01$; *** $p < 0.001$ vs. Oxaliplatin + PBS; by two-way ANOVA followed by Bonferroni's post-test.

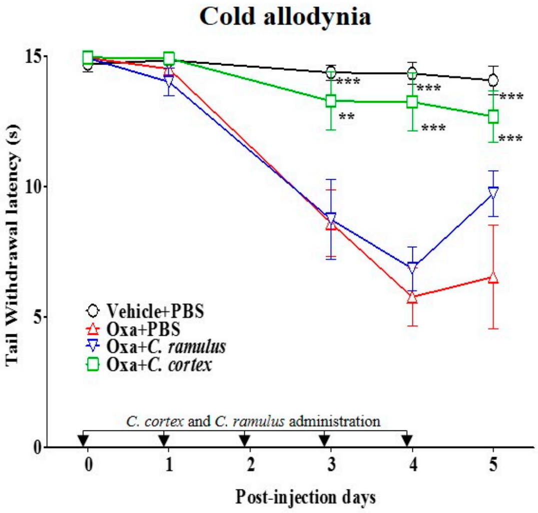

Figure S2. Effects of C. Cortex and C. Ramulus on oxaliplatin-induced cold allodynia in rats. Animals were randomly divided into 4 groups (*n* = 6/group), Vehicle + PBS, Oxaliplatin + PBS, Oxaliplatin + C. Ramulus, and Oxaliplatin + C. Cortex. Oxaliplatin or vehicle (5% glucose) was administered intraperitoneally on day 0. C. Cortex, C. Ramulus (200 mg/kg) or PBS was administered orally for five consecutive days after an oxaliplatin or vehicle injection. Data are presented as mean ± S.E.M.; ** $p < 0.01$, *** $p < 0.001$ vs. Oxa + PBS; by two-way ANOVA followed by Bonferroni's post-test.

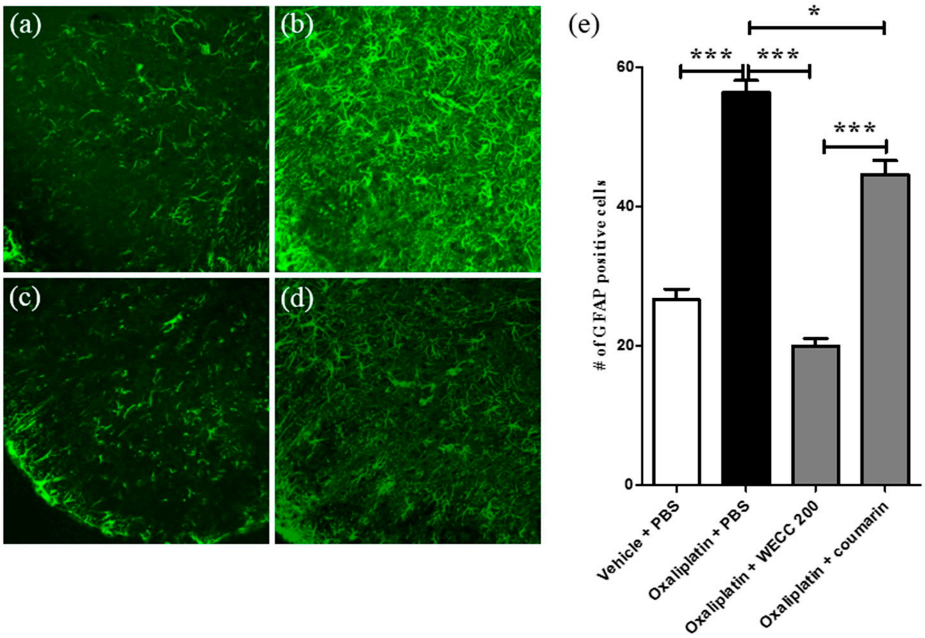

Figure S3. Immunohistochemical analysis of spinal astrocytes in the sacral spinal cord. Animals were randomly divided into 4 groups (*n* = 6/group), Vehicle + PBS, Oxaliplatin + PBS, Oxaliplatin + WECC 200, Oxaliplatin + coumarin. Representative images of GFAP positive cells (astrocytes) in the spinal dorsal horn of Vehicle + PBS (**a**); Oxaliplatin + PBS (**b**); Oxaliplatin + 200 mg/kg WECC (**c**); and Oxaliplatin + 10 mg/kg coumarin (**d**) groups; (**e**) Quantification results of GFAP positive cells in the four groups. *n* = 6 per group. Data are presented as mean ± S.E.M.; * $p < 0.05$; *** $p < 0.001$, by one-way ANOVA followed by Bonferroni's post-test.

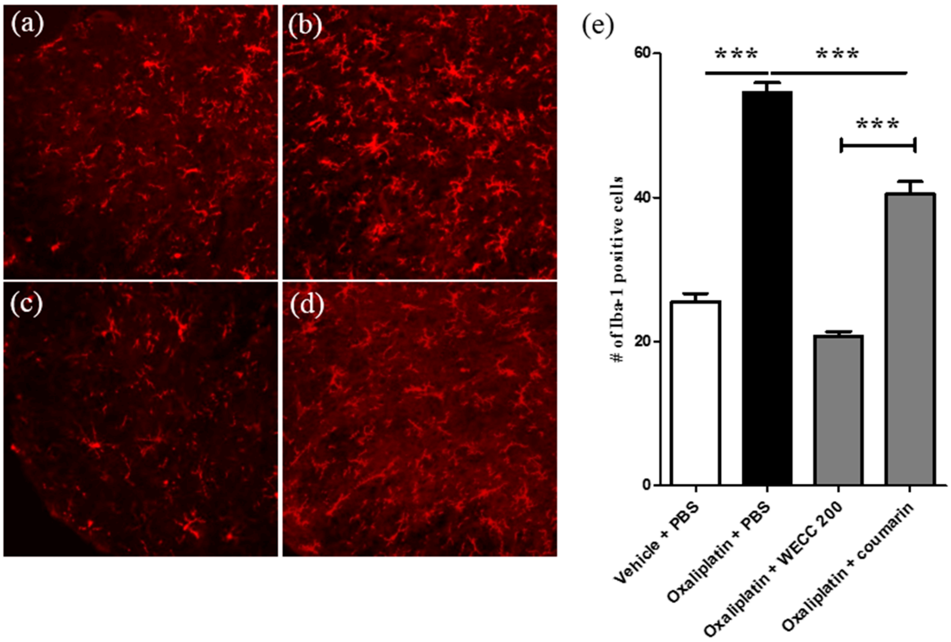

Figure S4. Immunohistochemical analysis of spinal microglia in the sacral spinal cord. Animals were randomly divided into 4 groups (*n* = 6/group), Vehicle + PBS, Oxaliplatin + PBS, Oxaliplatin + WECC 200, Oxaliplatin + coumarin. Representative images of Iba-1 positive cells (microglia) in the spinal dorsal horn of Vehicle + PBS (**a**); Oxaliplatin + PBS (**b**); Oxaliplatin + 200 mg/kg WECC (**c**); and Oxaliplatin + 10 mg/kg coumarin (**d**) groups; (**e**) Quantification results of Iba-1 positive cells in the four groups. *n* = 6 per group. Data are presented as mean ± S.E.M.; *** $p < 0.001$, by one-way ANOVA followed by Bonferroni's post-test.
